# Use of Email and Telephone Prompts to Increase Self-Monitoring in a Web-Based Intervention: Randomized Controlled Trial

**DOI:** 10.2196/jmir.1981

**Published:** 2012-07-27

**Authors:** Mary L Greaney, Kim Sprunck-Harrild, Gary G Bennett, Elaine Puleo, Jess Haines, K Vish Viswanath, Karen M Emmons

**Affiliations:** ^1^Center for Community-Based ResearchDana-Farber Cancer InstituteBoston, MAUnited States; ^2^Department of Psychology and and NeuroscienceDuke UniversityDurham, NCUnited States; ^3^Duke Global Health InstituteDuke UniversityDurham, NCUnited States; ^4^Department of Public HealthUniversity of Massachusetts AmherstAmherst, MAUnited States; ^5^Department of Family Relations and Applied NutritionUniversity of GuelphGuelph, ONCanada; ^6^Department of Society, Human Development, and HealthHarvard School of Public HealthBoston, MAUnited States; ^7^Department of Society, Human Development & HealthHarvard School of Public HealthBoston, MAUnited States

**Keywords:** Web-based health promotion intervention, self-monitoring, prompts

## Abstract

**Background:**

Self-monitoring is a key behavior change mechanism associated with sustained health behavior change. Although Web-based interventions can offer user-friendly approaches for self-monitoring, engagement with these tools is suboptimal. Increased use could encourage, promote, and sustain behavior change.

**Objective:**

To determine whether email prompts or email plus telephone prompts increase self-monitoring of behaviors on a website created for a multiple cancer risk reduction program.

**Methods:**

We recruited and enrolled participants (N = 100) in a Web-based intervention during a primary care well visit at an urban primary care health center. The frequency of daily self-monitoring was tracked on the study website. Participants who tracked at least one behavior 3 or more times during week 1 were classified as meeting the tracking threshold and were assigned to the observation-only group (OO, n = 14). This group was followed but did not receive prompts. Participants who did not meet the threshold during week 1 were randomly assigned to one of 2 prompting conditions: automated assistance (AA, n = 36) or automated assistance + calls (AAC, n = 50). During prompting periods (weeks 2–3), participants in the AA and AAC conditions received daily automated emails that encouraged tracking and two tailored self-monitoring reports (end of week 2, end of week 3) that provided feedback on tracking frequency. Individuals in the AAC condition also received two technical assistance calls from trained study staff. Frequency of self-monitoring was tracked from week 2 through week 17.

**Results:**

Self-monitoring rates increased in both intervention conditions during prompting and declined when prompting ceased. Over the 16 weeks of observation, there was a significant between-group difference in the percentage who met the self-monitoring threshold each week, with better maintenance in the AAC than in the AA condition (*P *< .001). Self-monitoring rates were greater in the OO group than in either the AA or AAC condition (*P *< .001).

**Conclusions:**

Prompting can increase self-monitoring rates. The decrease in self-monitoring after the promoting period suggests that additional reminder prompts would be useful. The use of technical assistance calls appeared to have a greater effect in promoting self-monitoring at a therapeutic threshold than email reminders and the tailored self-monitoring reports alone.

**Trial Registration:**

ClinicalTrials.gov NCT01415492; http://clinicaltrials.gov/ct2/show/NCT01415492 (Archived by WebCite at http://www.webcitation.org/68LOXOMe2)

## Introduction

Self-monitoring of behaviors and health measures such as diet, smoking, physical activity, and weight is a key behavior change mechanism. Adherence to self-monitoring regimens is associated with greater behavior change [1-6], weight loss [5,7-10], and long-term maintenance of weight loss [11,12]. However, a major challenge has been initiating and maintaining use of self-monitoring tools. Prior to the widespread availability of personal computers and mobile devices, most self-monitoring was done with paper and pencil. Technology offers excellent potential for increasing the ease and engagement of self-monitoring.

Participants in Web-based interventions who self-monitored their weight or physical activity, or both, have been shown to have greater success than those who do not [5,13,14]. However, the use of self-monitoring tools has been suboptimal. For example, in a Web-based worksite behavior change intervention, only 11% of participants (41/378 participants) used the self-monitoring tools [15]. Limited use and attrition are concerns in Web-based intervention studies requiring participant enrollment [16,17], as many of these interventions are designed for multiple visits and consistent use. Previous research suggests that email or telephone prompts may promote website use [15,18-20].

Despite its importance, there is limited information about the frequency of self-monitoring in Web-based interventions. This may be because process data are seldom reported [14,21]. Nonetheless, the current literature indicates that self-monitoring rates in Web-based interventions are low [14,22]. An evaluation of the HealthPartners 10,000 Steps online program, which included six promotional activities, found that although 74% of participants tracked their behavior at least once, only 9% tracked their steps weekly throughout the 21-week intervention [23]. Notably higher rates of self-monitoring have been reported in interventions that included in-person meetings with study staff or group sessions, or both [5,18,24,25], but the costs associated with in-person meetings substantially reduce the possibility of the intervention being sustained. Thus, the purpose of this study was to examine the feasibility of implementing a prompting intervention in a Web-based health promotion intervention.

## Methods

### Healthy Directions 2

The prompting study (hereafter referred to as substudy) described in this paper was a substudy of Healthy Directions 2, a randomized controlled trial of a multiple risk factor cancer prevention intervention conducted in two urban primary care health centers located in metropolitan Boston, USA. In Healthy Directions 2, patients from 33 participating providers were recruited and randomly assigned to study arms at the provider level. Enrollment eligibility included being a health center patient, being 18+ years of age, having a scheduled well visit or chronic disease management appointment, and being able to read English. Patients were ineligible if they had undergone cancer treatment in the previous year or had a diagnosis of dementia, blindness, neurodegenerative disease, or psychiatric illness (including substance abuse, psychosis, or schizophrenia in the previous 5 years).

The Healthy Directions 2 intervention targeted multiple cancer risk factors and was designed to (1) promote physical activity, (2) reduce red meat intake, (3) increase fruit and vegetable consumption, (4) promote daily multivitamin use, and (5) promote smoking cessation, as applicable. Intervention components included an endorsement of behavior change by the participant’s health care provider; materials delivered through the study website or in print, based on participant preference; intervention materials for participants’ friends and family members; and links to community-based resources. The materials emphasized the importance of consistent and continued tracking of health behaviors. The website included a user-friendly section where patients could self-monitor all targeted behaviors at once. Although daily self-monitoring was encouraged, the website allowed participants to enter data for the day they logged into the website and for the 2 days prior. After entering data, participants received immediate feedback in the form of graphs and descriptive text. Participants could also view their data over time, to assess overall progress. Intervention materials were available via the Web or as a print packet. When joining the study, each participant randomly assigned to the intervention received a bottle of multivitamins, a pedometer, intervention materials or logon information for the study website, and a US $5 gift card. The Healthy Directions 2 study was approved by the institutional review board at Harvard Vanguard Medical Associates.

### Recruitment

After completing recruitment for the parent study, we recruited an additional 100 participants for the substudy. This substudy was separate from the parent study. Eligibility requirements were the same as those for the parent study, plus the following additional criteria: (1) having an email address, (2) having the ability to access the Internet daily, and (3) being willing to receive the Healthy Directions 2 intervention via the Web only.

Recruitment for both the parent study and the substudy was the same. Eligible patients were sent an introductory letter that outlined the study and let them know that they may be approached and invited to join the study at their upcoming appointment. At check-in, study staff met the patient and verbally introduced the study, and interested individuals provided written informed consent and completed a self-administered baseline survey.

**Figure 1:**
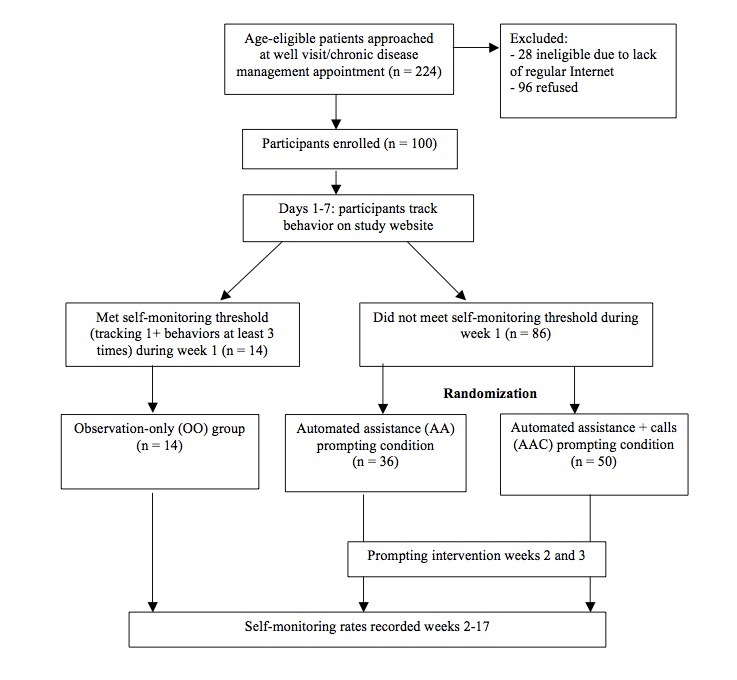
Flow of participants through the substudy.

During recruitment for the substudy, eligible participants were made aware that they may receive an additional intervention that would include emails and possibly two technical assistance calls. After completing the survey, each substudy participant received a bottle of multivitamins, a pedometer, login information for the study website, and a US $5 gift card for completing the survey. Recruitment for the substudy was limited to one site (8 providers) and took place in March 2010. As Figure 1 shows, 224 people were approached to join the substudy, 28 were ineligible, and 100 enrolled (96 declined; response rate: 51%). The substudy was approved by the institutional review board at Harvard Vanguard Medical Associates.

### Prompting Conditions

We set a minimum threshold of self-monitoring at least one behavior 3 or more times per week [13]. Participants who met this threshold during week 1 did not receive prompts (observation-only group [OO]; n = 14) but were followed throughout the study as a comparison group with the two prompting conditions. Participants who did not meet the self-monitoring threshold during week 1 were randomly assigned, based on primary care physician, following the randomization scheme of the parent study to receive one of 2 prompting interventions: automated assistance (AA) or automated assistance + calls (AAC). Participants were actively prompted during weeks 2 and 3, and frequency of self-monitoring was tracked from week 2 through week 17.

#### Automated Assistance

Participants assigned to the AA condition (n = 36) received 2 weeks of daily emails during the prompting period (weeks 2 and 3) that encouraged them to track their behaviors via the study website. Email messages changed daily and included a brief message about the benefits of self-monitoring and a hyperlink to the study website. Participants could choose to respond directly to the email with their tracking information instead of logging into the website; study staff uploaded emailed self-monitoring data to the study website. Participants also received two tailored self-monitoring reports: the first at the end of week 2 and the second at the end of week 3. Reports provided feedback to the individual about his or her frequency of tracking for each of the behaviors during the previous week. The reports mirrored the self-monitoring graphs available on the website; we hoped that seeing the graphs would encourage individuals who had not self-monitored to visit the website and track their behaviors. Reports were viewed as part of the prompting intervention. If participants did not self-monitor, their reports reiterated the information on the benefits of self-monitoring that was included on the daily emails and encouraged self-monitoring via the study website.

#### Automated Assistance + Calls

Participants randomly assigned to the AAC condition (n = 50) were sent the emails and tailored self-monitoring reports, detailed above, and received two technical assistance calls. The first call was made at the end of the first week of prompting (week 2) and the second call took place at the end of the second week of prompting (week 3). The calls, conducted by a trained health coach, were designed to be brief (less than 5 minutes) and focused on troubleshooting technical questions (eg, trouble logging in or how to self-monitor on the website).

### Measures

#### Tracking Measures

We created two categories of tracking measures. The first, self-monitoring measures, focused on the frequency of monitoring. We created the second, threshold measures, to examine the minimum weekly therapeutic threshold of self-monitoring 3 or more times per week. We used multiple measures to attempt to fully capture participants’ interaction with monitoring.

##### Self-monitoring Measures

We determined the total number of self-monitoring events (range 0–112). We then determined the total number of weeks during which participants self-monitored at least once (range 0–16), the greatest number of continuous weeks in which participants self-monitored (range 0–16), and the frequency with which participants self-monitored each week (range 0–7).

##### Threshold Measures

Using the frequency of self-monitoring each week, we determined whether participants met the weekly threshold each week (yes or no), total number of weeks during which participants met the threshold (range 0–16), and the greatest number of continuous weeks in which participants met the threshold (range 0–16).

#### Demographic Measures

The baseline survey included items to assess race, Latino ethnicity, marital status, frequency of Internet use, comfort level in using computers, and participants’ financial situation by asking participants to rate the “money situation” in their household (comfortable with extras, enough but no extras, have to cut back, and can’t make ends meet). Age, sex, and primary care provider was determined by data obtained from participants’ electronic medical records.

#### Behavioral Variables

All behaviors were assessed using validated measures [26-28]. The outcomes were dichotomized as to whether the participant met the recommendation for that specific behavior (75+ minutes of vigorous or 150+ minutes of moderate physical activity/week; 3 or fewer servings of red meat/week; 5+ servings of fruits and vegetables/day; a multivitamin 6–7 times/week; and not smoking).

### Analysis

We obtained descriptive statistics for key variables (using SAS version 9.1; SAS Institute, Cary, NC, USA). We used chi-square statistics and analysis of variance to assess differences in demographics and in meeting the behavioral recommendations between those who met and those who did not meet the weekly self-monitoring threshold of tracking at least one behavior 3 or more times during week 1 (OO vs AA and AAC). We used analyses of variance to assess differences between the three groups (OO, AA, and AAC) and the self-monitoring variables and the threshold variables.

To examine the impact of the prompting conditions from baseline through the prompting period, we tested a series of 2 (group: prompting conditions) × 3 (time: weeks 1–3) repeated-measures models. Separate models were used for each dependent variable (eg, self-monitored each week, met weekly threshold of tracking at least one behavior 3 or more times/week, or frequency of self-monitoring each week). We used binomial repeated-measures models for the dichotomous outcomes and general liner models for the continuous outcomes. The models were specified with a within-group factor of time and a between-group factor of prompting condition. We conducted similar analyses to examine the impact of the prompting conditions over the 16 observational weeks. We then included the OO group in the analyses and conducted repeated-measures models with 3 (group: OO, AA, and AAC) × 16 (time: weeks 2–17) and post hoc tests to explore any differences between the groups.

## Results

### Participants

The sample (N = 100) was 53% (53/100) male, with a mean age of 45.6 years, and was racially and ethnically diverse, with 37% (37/100) of the sample being black and 8% (8/100) being Latino or Latina. Most participants were college graduates (70/100, 70%) and reported frequent Internet use (see Table 1). Demographic or behavioral variables did not differ between the OO group and the combined AA and AAC conditions. Among participants in the AAC condition, 80% (40/50 participants) completed call 1 and 70% (35/50 participants) completed call 2. In total, 6 participants required technical assistance (needed a user name or password, had emails going to their spam filter, or were not sure what to do).

**Table 1:** Demographic characteristics of the study sample (N = 100).

Characteristic		Data
**Age (years)**		
	Mean (SD)	45.6 (14.9)
	Range	21–84
**Race/ethnicity, n (%)** ^a^		
	White	52 (52%)
	Hispanic	8 (8%)
	Black	37 (37%)
	Other	3 (3%)
**Sex, n (%)** ^a^		
	Male	53 (53%)
	Female	47 (47%)
**Education, n (%)** ^a^		
	High school diploma or less	5 (5%)
	Some college	24 (24%)
	College graduate or more	70 (70%)
Married, n (%)		63 (63%)
**Self-rated health status, n (%)** ^a^		
	Fair/poor	7 (7%)
	Good	44 (44%)
	Excellent/very good	47 (47%)
**Household financial situation, n (%)** ^a^		
	Comfortable, with some extras	49 (49%)
	Enough, but no extras	32 (32%)
	Have to cut back	15 (15%)
	Cannot make ends meet	4 (4%)
**Frequency of Internet use (/week), n (%)** ^a^		
	once	5 (5%)
	2–4 times	14 (14%)
	5+ times	80 (80%)
**Comfort level using a computer, n (%)** ^a^		
	Very uncomfortable/uncomfortable	7 (7%)
	Comfortable/very comfortable	91 (91%)

^a ^Due to missing values, percentages may not total 100%.

### Self-Monitoring Rates

Overall, 99.92% of participants who self-monitored, regardless of prompting condition and frequency of self-monitoring, tracked all four (nonsmokers) or five (smokers) of the targeted behaviors each time that they self-monitored (1219 of 1220 times tracked). In addition, during 76.8% (205/267) of the weeks when participants self-monitored at least once, they met the weekly threshold for self-monitoring during that week (tracking 1 or more behaviors 3 or more times). In all conditions, self-monitoring rates decreased over time.

#### Self-Monitoring in the Observation-Only Group

The OO group consisted of study participants who met the weekly self-monitoring threshold (tracking 1 or more behaviors 3 or more times) during week 1 and therefore did not receive prompts. Within this group, some self-monitoring occurred in 100% of the observational weeks.

**Figure 2:**
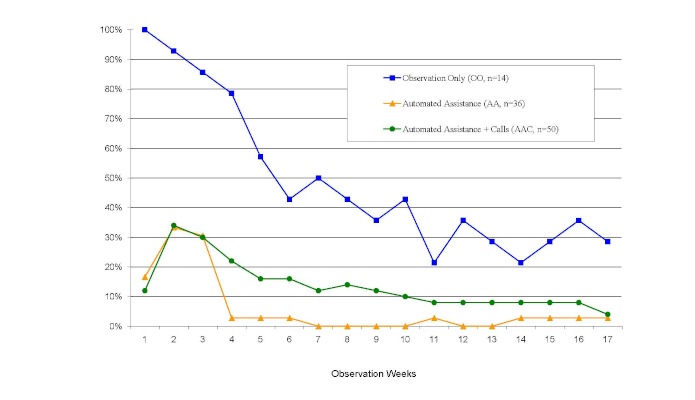
Percentage of participants, by group, self-monitoring 1 or more times per week (N = 100). The prompting intervention occurred during weeks 2 and 3. AA = automated assistance group, AAC = automated assistance + calls group, OO = observation-only group.

Figure 2 shows the percentage of participants within each group (OO, AA, and AAC) who self-monitored at least once during a week. As seen, the OO group had a greater percentage of people self-monitoring at least once per week than either the AA or AAC group. However, self-monitoring rates for all groups declined over time.

**Figure 3:**
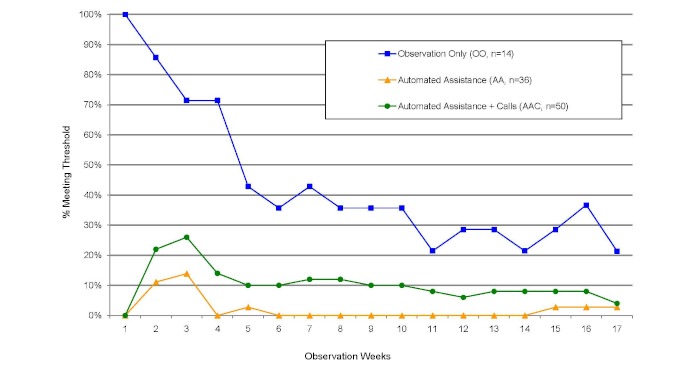
Percentage of participants, by group, meeting the self-monitoring threshold by week (N = 100). The prompting intervention occurred during weeks 2 and 3. The weekly threshold for self-monitoring was defined as tracking 1 or more behaviors 3 or more times during a week. AA = automated assistance group, AAC = automated assistance + calls group, OO = observation-only group.

Figure 3 shows the percentage of participants within each group (OO, AA, and AAC) who met the self-monitoring threshold during a week. As shown, between 21% (3/14) and 86% (12/14) of participants in the OO group met the threshold each week. The OO group had a greater percentage of participants who met the self-monitoring threshold each week than either the AA or AAC group. Self-monitoring in the OO group dropped most precipitously during the first 6 weeks; rates then stabilized, with around 30%–40% of this group continuing to track in the remaining weeks and a slightly smaller percentage meeting the weekly threshold.

#### Impact of the Prompting Interventions

##### During the Prompting Period

Time had a within-group effect on whether self-monitoring occurred each week (*P *< .001), on whether the threshold was met each week (*P *= .006), and on frequency of self-monitoring each week (*P *< .001). The between-group differences and group-by-time interactions were not significant.

##### Over the Course of the Observation Period

The repeated-measures models examining whether self-monitoring occurred each week revealed a within-group effect for time (*P *< .001) but not a between-group effect. The analyses examining whether the threshold was met each week revealed a within-group effect (*P *< .001) and a between-group effect (*P *= .009). The AA condition was less likely than the AAC condition to meet the threshold each week (odds ratio 0.27, 95% confidence interval 0.09–0.72). The group-by-time interaction was not significant. The analyses examining frequency of self-monitoring each week determined that there was a within-group effect (*P *< .001) but not a between-group effect.

In the AAC condition, there were more weeks when people self-monitored their behaviors at least once than in the AA condition (16/16, 100% vs 10/16, 63% weeks, Figure 2). Similarly, in the AAC condition at least one participant met the weekly threshold during a greater number of weeks than in the AA condition (16/16, 100% vs 6/16, 31% weeks, Figure 3). In addition, there was a significant difference between the AA and AAC conditions in the mean number of weeks during which participants met the threshold (AAC: mean 1.8, SD 3.9 weeks; AA: mean 0.4, SD 0.8 weeks; *P *= .04). There were, however, no significant differences between the two prompting conditions in the self-monitoring variables (frequency of self-monitoring, total number of weeks self-monitored, and greatest number of continuous weeks self-monitored) or in the greatest number of continuous weeks when the participants met the threshold.

#### Differences Between the OO and the Prompting Conditions

The results of the repeated-measures models and post hoc analyses examining the three self-monitoring outcomes determined that the OO group was significantly different from both of the prompting conditions on all outcomes (*P *< .001), with results favoring the OO group.

### Response to Email Prompts

Only a small percentage of participants (9/86, 10%) replied to the tracking reminder emails with their tracking information, but of those who responded, 67% (6/9) replied multiple times.

## Discussion

Consistent and continued self-monitoring is an instrumental strategy for initiating and maintaining behavior change [1-5,7-12], and technology provides a platform that enables participants to receive immediate, tailored feedback. Data show that 78.3% of the US population have access to the Internet [29], and that the use of Web-based interventions is increasing [17]. The Internet and Web-based interventions provide the opportunity to develop and implement behavior change interventions that actively promote self-monitoring. Nonetheless, self-monitoring rates remain low, and the potential for Web-based self-monitoring has not yet been realized, particularly in interventions that do not include in-person interaction with study staff (eg, health coaches). Providing assistance or prompts to encourage self-monitoring could be a key strategy leading to consistent and sustained self-monitoring [4,18]. Testing and developing sustainable strategies to initiate self-monitoring and to support consistent tracking is warranted.

The purpose of this study was to examine the effect of two limited prompting interventions on increasing participants’ use of self-monitoring tools available on the study website. In this study, participants who did not meet the self-monitoring threshold of tracking at least one behavior 3 or more times during the first week of the study were enrolled in one of two prompting conditions. Both prompting conditions yielded a modest but significant increase in self-monitoring each week (yes vs no), meeting the weekly threshold (yes vs no), and frequency of self-monitoring/week during the prompting period. After prompting ceased, self-monitoring rates decreased in both prompting conditions. Although the significant within-group effect remained throughout the observation period, the increased self-monitoring during the prompting period coupled with the subsequent decline suggest that reminder emails can increase prompting, but that additional subsequent email reminders may be useful to help sustain this increase. Future research is needed to examine the effectiveness for different prompting intervals [30].

Over the course of the study, a greater percentage of participants in AAC, the group that received technical assistance calls, met the weekly self-monitoring threshold, suggesting that brief contact, even in the context of technical assistance calls, may be beneficial for promoting and sustaining tracking. This limited contact with study staff may have increased motivation to track by removing technical barriers, or simply served as a gentle push to self-monitor. In total, 80% of participants in the AAC condition completed call 1, and 70% completed call 2; higher completion rates may have resulted in the AAC prompting intervention having a greater impact on self-monitoring. Future research is needed to explore the nature of interactions with staff that prompt higher levels of engagement (modality, frequency, content, etc), and whether a more explicit focus on enhancing motivation to track may yield greater self-monitoring.

Over the course of the study, the OO group had the highest rates of self-monitoring each week. However, even with this group’s strong start, their self-monitoring during weeks 3–5 declined precipitously, suggesting that this may be a critical time to intervene. Beginning with week 4, when prompting ended, self-monitoring rates decreased in both the AA and AAC groups. This general across-the-board attrition is similar to those seen in other studies [31,32] and is of concern.

It is noteworthy that participants who self-monitored chose to track multiple behaviors simultaneously 99.9% of the time. This is encouraging and suggests that tracking multiple behaviors is not burdensome. The willingness to track multiple behaviors in this study may have been due, in part, to the beta testing of the website that was conducted to inform a user-friendly design. The website was specifically structured so that all tracking could be completed on one page, thus making it easy to track all behaviors at the same time. In addition, it is striking that in 76.8% (205/267) of the weeks when participants self-monitored they met the threshold for that week, suggesting that specific strategies could be helpful for individuals who are willing to self-monitor but do not reach a weekly therapeutic threshold. The response rate to the tracking emails was low. It is possible that participants who were motivated to track simply logged in to the website and recorded their self-monitoring data themselves without assistance. It also could be that participants found it difficult to respond to the emails because of the timing of the sent messages (ie, unable to respond to the email during the workday). One Web-based study found that participants who actively responded to emails had a greater increase in fruit and vegetable intake than did participants who did not reply to emails [20].

Most often tracking has been limited to pencil and paper. Burke and colleagues reported that participants found self-monitoring via a personal digital assistant to be more socially acceptable than monitoring in paper logs [30]. The Healthy Directions 2 website was designed to be accessed via a computer, and if accessed via a smart phone, the user was required to login and would need to use the zoom capacity to clearly read text and enter self-monitoring data. Future Web-based interventions may want to consider ways that smart phones and other electronic devices can be used to allow participants to easily enter self-monitoring data that are uploaded to the study website.

Study limitations include a modest sample size; a largely well-educated sample, which may limit generalizability; and the lack of a nonprompting control group that included only participants who did not self-monitor during the first week of the study. Study strengths include having a diverse racial and ethnic sample and a design that allowed comparison with a group already motivated to record their behavior.

It would be helpful if interventions that include self-monitoring provided information about these rates or used consistent definitions of self-monitoring. But, to date, the literature has not provided either, making it difficult to compare adherence across studies [33]. In addition, research is needed to understand what external factors or personal attributes and characteristics motivate initial self-monitoring without prompting. Better understanding and use of these elements in interventions could be used to improve participation by less self-motivated populations. A 2009 literature review on the use of prompts in behavior change interventions concluded that tailoring of periodic prompts through regular contact with a counselor was associated with positive behavior change [29]. It may be that prompting interventions, including the prompting strategy, the frequency of contact, and the content of the message, should be individually tailored based on individual characteristics and behaviors that are assessed at baseline and throughout the intervention period. This will be an important area for future research.

The 2010 US Affordable Care Act emphasizes prevention and engaging patients as active participants in their health care [34,35]. Encouraging self-monitoring could play an important role in this effort, and comparing different prompting intervention to determine the most effective intervention is important. The use of technology-based tools that include feedback on progress and goals could make self-monitoring more accessible. However, little information is available about factors associated with self-monitoring, particularly self-monitoring conducted via personal computers and mobile devises, and less is known about best practices of using prompts to increase self-monitoring. Research is needed to understand what mechanisms can be used to increase use of self-monitoring tools to therapeutic levels. Different strategies may be needed to help individuals initiate and develop a strong foundation of self-monitoring and to maintain their motivation to continue tracking over long periods of time.

## Multimedia Appendix 1

Consort EHealth Checklist V1.6 [36].

## Abbreviations

AA: automated assistance group
AAC: automated assistance + calls group
OO: observation-only group
